# Capturing and Understanding the Dynamics and Heterogeneity of Gene Expression in the Living Cell

**DOI:** 10.3390/ijms21218278

**Published:** 2020-11-05

**Authors:** Amparo Pascual-Ahuir, Josep Fita-Torró, Markus Proft

**Affiliations:** 1 Department of Biotechnology, Instituto de Biología Molecular y Celular de Plantas, Universitat Politècnica de València, 46022 Valencia, Spain; 2 Department of Molecular and Cellular Pathology and Therapy, Instituto de Biomedicina de Valencia IBV-CSIC, 46010 Valencia, Spain; jfita@ibv.csic.es

**Keywords:** gene expression, transcriptional activation, transcriptional memory, single-cell variability, reporter assays, stress adaptation, transcriptional dynamics

## Abstract

The regulation of gene expression is a fundamental process enabling cells to respond to internal and external stimuli or to execute developmental programs. Changes in gene expression are highly dynamic and depend on many intrinsic and extrinsic factors. In this review, we highlight the dynamic nature of transient gene expression changes to better understand cell physiology and development in general. We will start by comparing recent in vivo procedures to capture gene expression in real time. Intrinsic factors modulating gene expression dynamics will then be discussed, focusing on chromatin modifications. Furthermore, we will dissect how cell physiology or age impacts on dynamic gene regulation and especially discuss molecular insights into acquired transcriptional memory. Finally, this review will give an update on the mechanisms of heterogeneous gene expression among genetically identical individual cells. We will mainly focus on state-of-the-art developments in the yeast model but also cover higher eukaryotic systems.

## 1. Introduction

**Figure 1:**
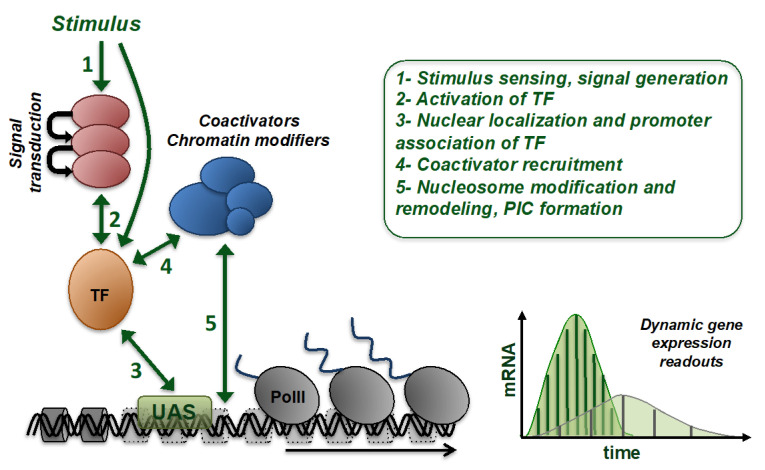
Determinants of dynamic transcriptional regulation of eukaryotic genes. Schematically are shown the processes that can modulate the timing and efficiency of activated gene expression. A given stimulus can give rise to different gene expression patterns in a gene- and cell-specific manner. TF, transcription factor, UAS, upstream activating sequence, PolII, RNA polymerase II, PIC, preinitiation complex.

The modulation of gene expression is a key feature of all living organisms and permits the adjustment of the protein composition of a cell in response to infinite environmental cues or during differentiation processes [1,2,3]. Traditionally, the expression rate of a gene has been measured by the quantification of its product, either mRNA or protein, using invasive methods, which generally impede the detection of the true dynamic nature of gene expression regulation. In the past decades, numerous approaches have been developed to study gene expression in real time and in the living cell by the introduction of different fluorescent or bioluminescent reporters or in situ hybridization techniques. This has greatly advanced our perception of the highly dynamic nature of transcriptional regulation [4,5]. It is clear now that the dynamics of transient gene expression is crucial for the appropriate cellular adaptation to changing environments [6], as is the timing and pattern of gene regulation fundamental for the fate of developmental programs [7]. In eukaryotic organisms, the activation of gene expression is a multistep process including transcription factor (TF) association with upstream control regions or enhancers, chromatin modification and remodeling, recruitment of co-activators and RNA polymerase, transcriptional elongation and termination, mRNA modification, and nuclear export. At each of these steps, gene expression dynamics can be modulated for specific genes. Please refer to excellent reviews for the mechanistic details of eukaryotic gene expression regulation in general [8,9,10,11], which is not the aim of this overview. In this review, we will focus on the molecular steps, from signal transduction to active gene transcription, which confer dynamic gene- and cell-specific regulation (Figure 1). We will summarize the mechanisms that modulate the expression rates of genes according to different physiological determinants of the organism and compare methods for the in vivo determination of dynamic gene expression.

## 2. Technical Approaches to Capture Gene Expression In Vivo

Traditional methods measure gene expression by the invasive determination of its final mRNA product by RT-PCR or Northern blotting or of the activity of a reporter protein such as β-galactosidase. Although, in principle, these techniques can infer the dynamics of gene expression in cell populations by serial sample preparation, the resolution and applicability to different parallel conditions of these approaches are limited. More directly, RNA polymerase II (PolII) association with target genes during activation can be dynamically estimated by kinetic chromatin immunoprecipitation (ChIP) techniques [12,13,14]. However, the comparison of transcriptional kinetics is very laborious with the ChIP approach.

Another way to quantify the dynamics of transcription is the direct visualization of the nascent mRNA by in vivo imaging procedures [15]. This was first established, together with other microscopic techniques, by Janicki et al. [16] in order to get a closer look at the true kinetics of the gene expression process in a living cell. One strategy to visualize mRNA molecules in vivo is the introduction of multiple sequence tags into the gene of interest, which form characteristic stem–loop structures in the corresponding mRNA molecule, recognized by detector proteins fused to fluorescent markers. Common detection systems rely for example on the specific recognition of tertiary RNA structures by the MS2 or PP7 bacteriophage coat proteins [17,18]. Instantaneous detection of nascent mRNA molecules by this method has enabled studies of transcription dynamics in living cells from bacteria to humans [19,20,21,22,23]. Very recent approaches combine in vivo labeling of DNA, nascent RNA, and a fluorescent protein to resolve the spatiotemporal process of transcriptional induction at a single locus [24]. Although these approaches are highly sensitive and are applicable at single-cell resolution, they require engineered genes for visualization and are generally time-consuming, which greatly limits the parallel study of several genes or their application to diverse conditions that modulate transcriptional dynamics.

A versatile way to quantify gene expression changes in vivo is the use of short-lived fluorescent proteins or luciferases in reporter gene assays. Although these approaches are not specific for transcription, they allow the continuous monitoring of gene expression in real time with little experimental effort. Typically, the fluorescent or bioluminescent protein is expressed from the control region of interest, either on plasmids or integrated in the genome, and protein synthesis is quantified over time by time-lapse microscopy, fluorescence cytometry, or fluorescence or luminescence readers. Fluorescent markers such as green fluorescent protein (GFP) are the most widely used indicators for dynamic in vivo gene expression studies [25,26,27] because of their high photon yield that is compatible with time-elapsed studies in single cells [28,29,30]. Additionally, GFP activity does not depend on additional cellular cofactors, and engineered versions with different colors are available for the simultaneous visualization of gene expression from several loci in the same cell [31]. However, several limitations exist that make fluorescent proteins a less suitable approach, especially for the faithful detection of transient and highly dynamic fluctuations of gene expression. Fluorescent proteins of the GFP family are very stable, which is a serial constraint for a good resolution of transient gene expression. Therefore, destabilized GFP variants have been developed [32,33,34], which, however, still show considerable half-lives of at least 30 min and loss of signal output [35]. Furthermore, GFP shows a slow maturation that is an additional obstacle for the real-time visualization of fast transcriptional responses. Furthermore, the continuous monitoring of fluorescent protein activity requires external excitation with high-energy light sources, which causes problems of autofluorescence and phototoxicity.

Many of these inconveniences of fluorescent markers for gene expression studies can be avoided by the use of bioluminescent proteins such as luciferases as reporters. Luciferases do not require external excitation and are co-translationally active. Additionally, unstable versions of firefly and other luciferases have been created for time-elapsed gene expression studies, from bacteria to human cells [36,37,38]. The destabilization of the firefly luciferase by the addition of combined protein and mRNA degradation motifs has been especially useful in the yeast model, where these tools allow real-time monitoring of gene expression fluctuations with unprecedented resolution [39,40,41,42]. Although luciferase reporters have significantly lower signal levels and are normally used in cell populations, continuous single-cell gene expression studies are technically possible with unstable luciferase reporters in yeast cells [42]. Bioluminescent reporters, however, pose yet other technical challenges for the continuous measurement of gene activity over time. The specific substrate for the luciferase enzyme, e.g., luciferin in the case of the firefly enzyme, has to be provided externally at sufficient concentration for long-term studies. Also, luciferase activity depends on oxygen and ATP levels, which might additionally have an influence on the light emission levels obtained during prolonged live cell assays.

The techniques described above enable researchers to quantitate dynamic gene expression events by measuring the final product of the process. In the past years, also the visualization of individual TFs and their dynamic interaction with genomes and *cis*-regulatory elements at the single-cell and single-molecule levels has greatly enhanced our models of eukaryotic gene activation [43,44].

## 3. Activated Gene Expression: A Highly Dynamic Dose-Dependent Biological Function Sensitive to Many Physiological and Genetic Factors

**Figure 2:**
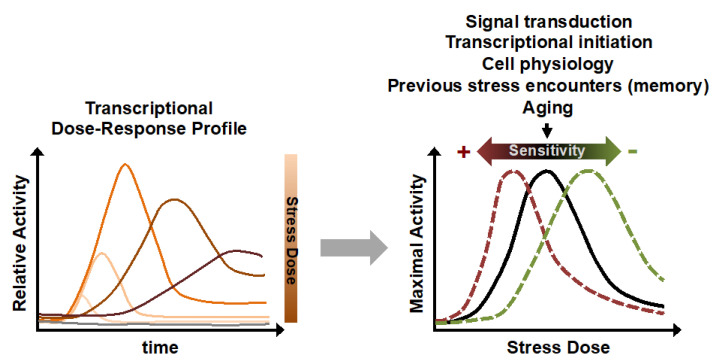
Transcriptional dose–response profiles and their modulation. Continuous monitoring of gene expression quantifies the response of a cell population for a specific gene at any possible stress dose (dose–response (DR) profile, depicted on the left). The responsiveness of gene expression over a stress gradient can dynamically change, as shown on the right. For different genes, the sensitivity of the dose–response might depend on differential signal transduction or on the ability of each gene to engage in active transcription. The same gene might show different DR profiles in cells with altered physiology, different age, or after repeated stress responses.

Gene expression changes upon external stimuli often affect many genes simultaneously in order to adapt the organism to the changing environment. Transient transcriptional activation is largely dependent on the strength of the stimulus or stress. Recent applications of time-elapsed luciferase reporters have revealed the dose-dependent dynamics of these responses by simultaneously measuring gene expression upon continuously increasing stimulation [39,45]. The resulting dose–response (DR) profiles contain information about how cells adjust gene expression upon dynamic environmental signals (Figure 2) [46]. A typical DR profile shows very little transcriptional activation upon low threshold stimulation, which is continuously increased until reaching a maximal transcriptional output upon the optimal stimulation in the dynamic range. Further increases in stimulation do not further enhance gene expression but in many occasions lead to delayed and/or less efficient expression due to inhibition of the gene expression process by harsher induction conditions. It is important to note that an apparently low transcriptional response in cell populations can be produced when only a fraction of the cells actually respond to the stimulation. This phenomenon called bimodal gene expression has been found in several transcriptional responses to environmental changes and occurs often at threshold stress levels [47,48,49].

There are many factors which modulate the DR profile of the same or of different genes and cause shifts towards more or less sensitive behaviors (Figure 2). One is the intrinsic promoter structure. Quantitative studies in yeast populations have shown that different promoters have distinguishable sensitivities towards the same abiotic stresses such as osmotic or oxidative challenges, resulting in different half-maximal stimulus concentrations [45]. It has been recently elucidated that the dynamic of a transcriptional stress response is modulated at both levels of transcription rate and duration and that the gene expression output is modulated at different stages by multiple genetic determinants [50]. Detailed studies of specific inducible yeast promoters have been undertaken successfully to improve the accessibility and affinity of TF binding sites in artificial promoters to create gene expression responses with altered dynamics [51,52,53].

The ability to study transcriptional regulation at the single-locus level has advanced our model of how genes switch from an inactive to an active state in a stochastic manner [54]. This phenomenon is called transcriptional bursting and describes how an inactive locus can switch to the active synthesis of active nascent RNA for a limited period, allowing transcription of several RNA polymerases simultaneously. Both the timing and the frequency of the burst can be subject to regulation, thereby adjusting gene expression to specific stimuli. The transcriptional burst itself is not a subject of this review, and the interested reader is referred to excellent recent review articles [55,56]. Here, we are interested in the molecular mechanisms which favor or disfavor bursting at specific genes. The critical limiting steps prior to the formation of the preinitiation complex (PIC) and transcriptional initiation are transcriptional activator binding and chromatin and nucleosome remodeling. Large-scale mutagenesis approaches using yeast promoters confirmed that indeed the upstream regulatory sequence is generally responsible for high transcriptional activation [57]. Complementary to these studies, the introduction of nucleosome disfavoring sequences in promoters largely favored active gene expression [58], and modulating the extension of nucleosome-free regions is an important manner to dynamically tune the transcriptional activity of a genetic locus [59]. This indicates that facilitating a stable activator contact with its cognate promoter DNA is one of the rate-limiting steps in dynamic transcriptional activation. Systematic studies in yeast have confirmed this and shown that in most cases, the amount of activator protein, rather than the amount of binding sites, is the major determinant of gene expression rate [60]. Studies of individual yeast TFs at the single-molecule level revealed that unspecific target binding is common for different TFs, while coordinated chromatin remodeling is necessary for dynamic TF association and transcriptional bursting [61,62]. In general, the timing of transcriptional initiation and the efficiency of mRNA production seem to be linked to different recycling dynamics of TFs [63]. Very recently, the application of simultaneous visualization techniques for TF binding and RNA production at a single locus has permitted to directly correlate Gal4 activator binding with the amount of RNA produced from different Gal4-regulated genes in a process which is highly dependent on the presence of nucleosomes [64]. Single-molecule experiments at the *GAL1*/*GAL10* locus furthermore indicated that transcription of non-coding (nc)RNAs can dynamically regulate gene expression and adjust different activation thresholds [65]. Also, for metazoan genes, the efficient recruitment of transcriptional activators and coactivators proximal to the transcriptional start site seems to be essential for dynamic gene activation. This has been reported for the proto-onco gene *cFos* and for a glucocorticoid-regulated reporter gene [66,67]. Specific mammalian TF levels are critical for creating dynamic transcriptional behaviors for various genes [68,69]. Additionally, nuclear architecture is an important determinant for target finding for individual TFs, according to single-molecule tracking experiments [70]. Generally, also in higher eukaryotic systems, the number and affinity of *cis*-regulatory elements in promoters is decisive for the dynamics of transcriptional bursting [71,72]. Stem cell bursting dynamics have been recently found to be regulated by a combination of chromatin-remodeling complexes and signal transduction pathways [73]. For developmental gene regulation in *Drosophila*, it has been recently shown that dynamic morphogen gradients modulate different bursting parameters to ensure graded mRNA synthesis at single loci during embryo development [74,75].

Chromatin remodeling is often the rate-limiting process affecting the transcription rates of eukaryotic genes [76]. Since the cell can keep different chromatin states or marks at specific genomic regions, these elements are important both for the short-term activation of environmental stress-responsive genes (discussed here) and for long-term memory effects (discussed in the next chapter). The requirement for nucleosome remodeling to ensure activator binding and/or stable PIC formation might be very variable at different inducible loci or for different physiological states of the cell. Thus, the efficient switch from an inactive to an active chromatin state modulates transcriptional dynamics in an important manner [76,77]. In yeast, several inducible genes such as *PHO5*, *INO1*, *GAL1*, *SUC2*, *GRE2*, *CUP1*, *HO* and others have been studied in detail to understand how the timely recruitment of co-activator complexes, which modify and remodel nucleosomes at the promoter chromatin, promotes dynamic transitions to the on state of genes responsive to diverse environmental stimuli [78,79,80,81,82,83]. In many cases, a functional interplay between different complexes such as SWI/SNF2, ISWI, CHD, INO80 ATPase-containing remodelers and SAGA, Rpd3 histone modifiers, or the mediator complex has been documented [78,79,84,85,86,87,88,89]. It is important to note that the time needed for efficient PIC formation after the first stimulation spans a wide range, from one or few minutes (typically, at acute stress-responsive genes) to hours (at nutrient-responsive or developmental genes). For example, a first induction of the nutrient-responsive *GAL1* gene is slow and requires intensive chromatin modifications for active Gal4 binding, PIC assembly at the promoter, and dynamic initiation [90,91,92]. This also leads to a pronounced bimodal expression at medium or low galactose concentrations, which is dependent on chromatin remodeling activities [90,93]. As a result, *GAL1* and similar slow-responding genes are prone to faster activation upon repeated stimulation by epigenetic and other mechanisms, as discussed in the next chapter. On the other hand, fast-responding stress genes such as the *GRE2*, other osmostress-responsive genes, or genes responding to xenobiotic insults are characterized by the rapid engagement of a pre-bound TF in PIC formation upon co-activator recruitment [88,94,95]. Accordingly, those genes do not show or have very little positive memory upon repeated exposure to the stressor [90,94].

Looking more upstream from transcriptional initiation, it is important to understand the dynamic signaling events which further increase the sensitivity of gene expression. External and internal signals are usually transmitted to specific TFs either directly or indirectly via signal transduction pathways. Frequently, activation derives from the direct binding of the TF to chemical compounds such as hormones, metal ions, or xenobiotics or from a post-translational modification of the TF, such as phosphorylation. As a result, the activated TF can acquire one or more of the following functionalities: enhanced nuclear retention and/or enhanced association with the cognate DNA motif, weakening of inhibitory factors, and favored recruitment of chromatin-modifying coactivators and PolII complexes. It is important to note that the dynamics of gene induction upon a given stimulus depends on how efficiently the TF is responsive to the stimulation. The same stress might provoke different gene expression outputs depending on the specific TF involved. For example, various transcriptional activators participate in the yeast osmostress response, and all receive a phosphorylation signal from the upstream stress-activated protein kinase (SAPK) Hog1, upon osmotic stress. Here, the TF Sko1 has been found to more sensitively activate gene expression as compared to several other TFs [46], which might enable the cell to adapt to a stress with a hierarchical response, employing differentially sensitive transcriptional activators. Other examples are xenobiotic binding TFs of the Pdr family in yeast, which very recently have been shown to transmit different gene activation dynamics by the distinguishable recognition of chemically divergent compounds [94]. On the other hand, an opposing, signal-integrating function has been described for the general stress-responsive transcriptional activator Msn2, which is able to process up to four different stress inputs into distinguishable dynamic transcriptional outputs based on its regulation of nuclear import and export [96,97,98].

To fully understand how cells respond to environmental cues by modulating gene expression, it is important to consider cells’ physiology. One has to keep in mind that cells in many cases engage in activated gene expression in order to overcome a particular disruption of their homeostasis and eventually return to the equilibrium state. The amount of gene regulation needed for this compensation is often dependent on the physiological properties of the cell. A simple example illustrates this dependence. It has been shown that yeast cells adapted to rich growth conditions tend to transcriptionally respond to abiotic stresses at lower doses, which could be explained by a more repressed general stress defense as compared to cells adapted to minimal growth conditions [46]. In the same vein, osmotic stress causes transcriptional adaptation at significantly lower stress concentrations in galactose- as opposed to glucose-grown cells, because galactose metabolism does not allow an efficient osmoprotection in budding yeast [99,100]. These examples illustrate that the physiological properties of a cell are important modulators of the sensitivity and dynamics of its transcriptional response to many stressors.

Cellular aging is a specific physiological change that has consequences for the dynamics of gene expression [101]. Work in the yeast model has shown that cell-to-cell heterogeneity of gene expression, also called transcriptional noise, increases in aged cells due to the lower expression of histone genes [102]. This global deregulation of precise gene expression control is manifested by the overall loss of promoter nucleosomes with advanced age [103]. Further studies revealed that the maintenance of a specific histone mark—H3K36 methylation—was critical to avoid transcriptional leakage during aging [104]. These data suggested that global changes in chromatin structure occur in old yeast populations, leading to an increase in transcriptional noise and a decrease in transcriptional fidelity [105]. However, by monitoring specific reporter genes in single yeast cells, noise reduction during normal aging has been reported, with a complete deregulation at the final stages of the aging process [106]. Also in individual mammalian cells, a great increase of transcriptional diversity has been found during aging [107,108,109,110,111]. Additionally, chromatin rearrangements of different magnitude have been reported in old mammalian cells and lead to altered gene expression dynamics for several genes [112,113,114,115,116]. Another important change in gene expression dynamics occurs in old cells at the level of rapid gene activation upon stress signals. Aging yeast populations show an increasing loss of efficiency, dynamics, and timing of oxidative stress-responsive reporter genes [46]. In T cells from old mice, the immune response is less tightly regulated, and an inefficient and heterogeneously uncontrolled transcriptional response has been found [117]. These data indicate that the loss of a timely and efficient transcriptional activation upon cellular stress might contribute to aging-related cellular and organismal dysfunctions [101,118,119].

Another important factor which shapes the transcriptional response to a given stimulus is cells history. Very often, a previous encounter with the same or related stress determines the transcriptional response in a later, repeated encounter. An improved response by means of transcriptional memory, as detailed in the next chapter, is only one of several scenarios. Indeed, transcriptional memory can be separated from acquired tolerance mechanisms in yeast, and both contribute to an enhanced fitness after a previous stress insult [120]. Furthermore, the prevalent behavior depends on the combination of different stress experiences. For example, repeated oxidative stress in yeast leads to a more efficient transcriptional activation [46,120], while repeated salt stress makes cells less responsive [90,121]. A combination of both stresses induces a mixed behavior, with cells responding less at low doses and more efficiently at high stress doses [46]. Additionally, it has been reported that a first round of xenobiotic-induced gene expression is very efficient in yeast cells, while a shortly repeated stimulation leads to a significantly reduced transcriptional activation [94]. This behavior can be explained by the rapid degradation of the main xenobiotic-regulated TF, Pdr1, after its activation [94].

## 4. Epigenetic Transcriptional Memory: Modulating Gene Expression Dynamics upon Repeated Stimulation

**Figure 3:**
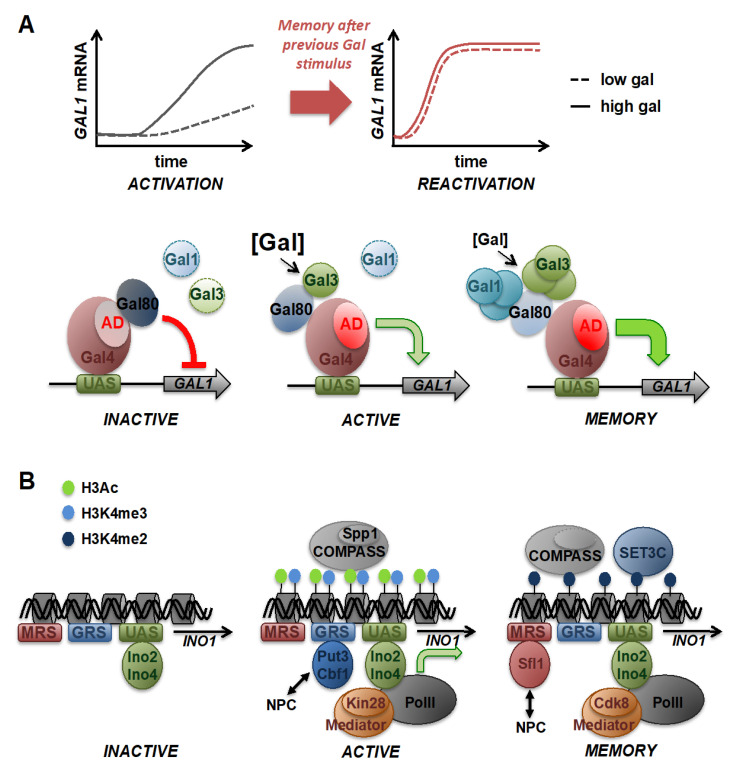
Mechanisms of transcriptional memory. (**A**) *Trans* mechanisms of improved reactivation at the *GAL1* gene after repeated galactose stimulation. Upper panel: Naïve cells exhibit slow and galactose-insensitive *GAL1* activation kinetics. After a previous encounter with galactose, cells respond with a faster, more efficient and sensitive gene induction. Lower panel: Details of transcriptional memory at *GAL1* by the reinforcement of signaling. The inactive *GAL1* gene is bound by the Gal4 transcriptional activator, which is completely inactivated by association with the Gal80 repressor masking the Gal4 activation domain (AD). A first round of *GAL1* transcriptional activation (active) is slow and inefficient due to the low amount of the Gal3 galactose sensor and signal transducer. After memory, the previously induced and inherited Gal1 and Gal3 proteins prepare the experienced cells for fast reactivation even at low galactose doses. Both Gal1 and Gal3 can induce *GAL1* expression by Gal80 inhibition. (**B**) Epigenetic mechanisms of memory at the *INO1* gene after repeated inositol starvation. The inactive *INO1* gene is characterized by absent histone acetylation or methylation despite Ino2,4 activator binding. A first round of *INO1* induction includes the physical anchoring of the gene to the nuclear pore complex (NPC) through binding of Put3 and Cbf1 to the *INO1* gene recruitment sequences (GRS). Histones are modified by acetylation and trimethylation of histone H3 by the Spp1-containing COMPASS complex. Transcriptionally competent RNA polymerase II is present, accompanied by the Kin28-containing mediator complex. After addition of inositol, the *INO1* gene shifts to the memory mode, which is characterized by prolonged NPC anchoring via Sfl1 and the *INO1* memory recruitment sequence (MRS), loss of Spp1 from COMPASS, predominant histone H3 dimethylation, binding of the SET3C histone deacetylase complex, and the association of a poised, transcriptionally inactive version of PolII sustained by the Cdk8 module of Mediator. *INO1* in the poised chromatin state is reactivated with faster kinetics.

An important mechanism that changes the expression dynamics of genes is epigenetic transcriptional memory. This phenomenon has been found in many organisms and describes the fact that a response to a previous environmental stimulus alters the dynamics of gene expression upon subsequent stimulation [122,123]. These heritable changes normally last during several cell generations and might permit the organism to respond faster and more efficiently to periodically occurring environmental challenges. For example, human cells memorize past infections by favoring interferon-γ-mediated gene induction [124], and plants show an enhanced transcriptional response to heat shock several days after a previous heat stress [125,126,127]. Budding yeast has been used very extensively in transcriptional memory investigation, because several nutritional changes induce profound memory effects, which can be easily studied in depth in this organism. Two genetic systems and environmental conditions have been mainly applied in yeast: the induction of the *GAL* genes (encoding the enzymes necessary for galactose utilization), which show strong epigenetic memory upon repeated exposure to galactose, and the expression of the *INO1* gene (encoding inositol-1-phosphate synthase), which is activated by inositol starvation and potentiated upon previous inositol deprivation (Figure 3).

In naïve yeast cells, induction of the *GAL* genes is slow and requires high inducer concentrations to be efficient. After a previous galactose encounter, this response is significantly faster and more sensitive to low galactose concentrations, and this memory state is maintained as long as through seven generations [90,91,128]. Different molecular mechanisms have been identified so far to explain the establishment of *GAL* memory. One mechanism is chromatin-based and leads to the relocalization of the *GAL* gene to the nuclear periphery, where it physically associates with the nuclear pore complex (NPC) upon the first induction [128,129]. The NPC subunit Nup100, the H2A.Z histone variant, and specific *GAL1* promoter sequences are important for this translocation [129]. *GAL1* tethering to the nuclear periphery is maintained even in the absence of active *GAL* gene transcription. However, disruption of *GAL1* interaction with the NPC did not affect *GAL1* transcriptional memory, which implies other memory mechanisms in the case of *GAL* genes [129]. Accordingly, it has been suggested that enhanced *GAL* induction during memory largely depends on the accumulation of signaling molecules, such as the Gal1 and Gal3 proteins [90,130,131] (Figure 3). Both factors bind to the Gal80 repressor [132], which inhibits *GAL* gene expression by masking the activator domain of the Gal4 transcriptional activator. Therefore, previous induction of Gal1/Gal3 can promote a faster activation by more efficiently counteracting *GAL* repression through reinforced signaling during memory. Indeed, the degree of *GAL* memory seems to correlate with the expression levels of Gal1 or Gal3 [90,130,133].

A more pronounced role for chromatin modification and relocalization to the NPC has been found in the case of memory at the *INO1* locus (Figure 3). Once activated by a first inositol deprivation, *INO1* adopts a memory-specific chromatin configuration characterized by the binding of a specific TF, the persistent translocation to the nuclear envelope, the incorporation of histone variants and histone modifications, and the recruitment of remodeled chromatin-modifying complexes and of a poised PolII complex [122]. The conversion from the activated to the memory state at *INO1* is mediated by the binding of the TF Sfl1 to a specific DNA motif in the promoter region, the so-called memory recruitment sequence (MRS) [134]. Both Sfl1 and MRS are necessary to keep *INO1* anchored at the nuclear periphery and to maintain several characteristic chromatin features. One feature is histone H3 dimethylation at lysine 4 (H3K4me2) at the *INO1* promoter and coding region [135]. Additionally, the histone variant H2A.Z is specifically incorporated into *INO1* chromatin during the memory phase [128,136]. H3K4me2 is produced by a remodeled version of the Set1/COMPASS complex lacking the Spp1 subunit necessary for H3K4me3 only during activated *INO1* transcription [134]. H3K4me2 is then recognized and maintained by the SET3C histone deacetylase complex, which is required to recruit a poised version of PolII to *INO1* during memory. PolII assembly during memory is different from PIC formation during normal gene activation, as it recruits PolII in a transcriptionally inactive form. This seems to be assured by PolII assembly in the absence of the Cdk7/Kin28 C-terminal domain (CTD) kinase [134]. Cdk7 promotes promoter escape of the enzyme during normal activation by PolII CTD phosphorylation, which is blocked during *INO1* memory. Additionally, Mediator binds as a specific, Cdk8-containing module, which might facilitate maintaining a poised PolII complex at the *INO1* locus during memory [134]. Altogether, the *INO1* memory mechanism shows that specific genes can adopt a memory configuration after a first stimulation via specific TFs, which is characterized by a combination of histone marks, NPC anchoring, and association of specific chromatin-modifying complexes leading to the permanent binding of PolII in a poised state, thus allowing a faster reactivation of transcription in repeated rounds of stimulation [122,137]. It is important to note that these findings from yeast research reflect several mechanisms conserved for epigenetic memory in higher eukaryotic organisms. Prolonged H3K4me2 is an epigenetic hallmark for memory also for plant and human genes [124,138,139,140]. Moreover, the persistent binding of a poised, transcriptionally inactive PolII has been found in *Caenorhabditis elegans* and human cells at some memory genes after previous stimulation [134,135,141].

## 5. Gene Expression Heterogeneity in Individual Cells

Genetically identical cells can show considerable variability in their gene expression patterns, a phenomenon generally called gene expression noise [142]. There are extrinsic and intrinsic factors causing a noisy expression across individual cells. Extrinsic sources of noise are, for example, the stochastic initiation of different transcriptional cell fate programs in multicellular organisms in response to gradients of morphogens [143,144]. Another example of the extrinsic induction of heterogeneous expression patterns is the response to inflammatory signals in mammalian organisms, where oscillations in NF-κB TFs determine different immune responses and specific cytokine production [145,146]. Furthermore, very recently it has been proposed that intrinsic stochasticity of gene expression favors plasticity and robustness of mouse stem cells [147].

Here, we will focus on the establishment and regulation of intrinsic noise of gene expression, which is originated by natural fluctuations of cellular and, especially, intra-nuclear factors or chromatin states [148,149]. In microorganisms, the stochastic heterogeneity of gene expression is a mechanism to improve the fitness of a cell population upon unpredicted environmental cues [150,151,152], in a process which has been coined “bet hedging” [153,154,155]. Similarly, cancer cell subpopulations with specific chromatin and gene expression variability are sources of drug tolerance during pharmacological treatment [156,157,158]. It is important to note that, in most cases, the observed heterogeneity is transient and reversible, because it is caused by epigenetic alterations or fluctuations in the activity of proteins critical to the process of gene expression [142,149,159,160,161].

Pioneering experiments exploring lac repressor regulation by the expression of fluorescent protein markers in bacteria discovered a striking cell-to-cell variability even in this simple regulatory circuit [142]. As a first intrinsic determinant of the observed noise, the abundance of the lac repressor itself was identified [142]. Similar studies in the yeast model using the inducible *PHO5* system confirmed gene expression heterogeneity in eukaryotic cells, which is modulated by extrinsic and intrinsic factors [159]. Later genome-wide studies revealed that low expression levels are generally accompanied by high noise, while highly expressed genes are not [162]. Similarly, housekeeping genes are usually more uniformly expressed among cell populations, while environmental stress-responsive genes show a noisier expression pattern [163,164,165]. This might be explained by the fact that fluctuations of housekeeping proteins is less compatible with essential cell functions as compared to stress-responsive proteins, whose fluctuations might be beneficial for the adaptation to environmental changes [166,167].

Several studies have addressed the question whether transcriptional noise was created by specific cellular or nuclear processes. Promoter engineering in bacteria has shown that variations in the intrinsic promoter design modulates the degree of noise [168], which suggests that altered affinity of TFs is a main source of transcriptional variability [169,170,171]. Indeed, the TF resources of a cell seem to be critical for noise determination, which increases with limited amount of active TF [172]. Recent studies point to the importance of different bacterial sigma factors in the modulation of transcriptional noise [173]. Studies of the yeast *PHO5* nucleosome positioning further demonstrated that specific chromatin configurations can be an important intrinsic source of transcriptional noise [174,175]. Genome-wide studies in single mammalian cells showed, on a broader scale, that changes in chromatin accessibility generally control transcript variability [176]. Specific histone marks such as H3K79 methylation have been furthermore shown to determine the level of expression noise [177]. Additionally, *Drosophila* genes with pre-bound, paused RNA PolII have been associated with a more uniform expression pattern, while disruption of PolII pausing increased the stochasticity of gene expression [178]. Transcriptional variability can furthermore be modulated by more physiological characteristics of the cell such as the cell cycle [179] or the process of mRNA export and nuclear compartmentalization, which can have functions in buffering stochastic transcription bursts [180].

It remains an intriguing question whether and how cells are able to regulate general or gene-specific transcription variability according to developmental or environmental changes. It has been determined that noisy gene expression is a complex heritable trait [181]. However, recent work in yeast identified a specific regulator, the methyltransferase Hmt1, as a master regulator of noise [182]. Hmt1 has been proposed to reduce transcriptional variability by methylating strategic effectors with functions in chromatin remodeling (Snf2) or translation (Rps2). Environmental stress could inactivate this pathway and increase cell-to-cell variability in order to better adapt to adverse conditions [182].

## 6. Conclusions and Future Perspectives

In the past years, we have witnessed many significant advances in the field of dynamic transcriptional responses. We have successfully moved from a static view of transcription towards a fully dynamic description of gene activation, both at the cell population and at the single-cell level. In fact, any transcriptional regulation cannot be sufficiently understood on the basis of fixed snapshots of the process. Many technical advances, from the continuous monitoring of gene expression by fluorescent and luminescent reporters to the single-cell tracing of TF binding, nascent mRNAs, and chromatin remodeling at single loci, have largely increased the resolution with which we are able to look at specific dynamic features of gene activation processes. TF dynamics and recycling, as well as specific chromatin remodeling have emerged from those studies as modulators of dynamic gene expression. A main future challenge, however, will be to link dynamic transcriptional readouts with higher-order determinants, such as nucleosomal or chromosomal topology and arrangement. Additionally, the great variety of changes in gene expression dynamics must have fundamental biological functions, which in many cases are not well known. For example, it will be important to reveal the molecular drivers of the loss of dynamic gene activation in aged cells, if and how transcriptional dynamics and memory change during adaptation to specific environments or during evolution, or how modulation of transcriptional heterogeneity is achieved and possibly impacts on adaptation and fitness of the cell.

## Abbreviations

TF	Transcription factor
PolII	RNA polymerase II
ChIP	Chromatin immunoprecipitation
GFP	Green fluorescent protein
NPC	Nuclear pore complex
PIC	Preinitiation complex
MRS	Memory recruitment sequence
GRS	Gene recruitment sequence
AD	Activation domain
DR	Dose–response
SAPK	Stress-activated protein kinase
CTD	C-terminal domain
